# Supplementary material for: End Sequence Analysis Toolkit (ESAT) expands the extractable information from single-cell RNA-seq data
Source: Genome Res. 2016 Oct;26(10):1397–410. doi: 10.1101/gr.207902.116 (PMC5052061; doi:10.1101/gr.207902.116)
Supplement: Supplemental Material [file supp_gr.207902.116_Supp_Materials.tar.gz › ESAT-esat_v0.1/doc/html/classjaligner_1_1_alignment-members.html]

ESAT: Member List


ESAT


- Main Page
- Related Pages
- Packages
- Classes
- Files

- Class List
- Class Index
- Class Hierarchy
- Class Members

All Classes Namespaces Files Functions Variables Enumerator Pages

- jaligner
- Alignment

jaligner.Alignment Member List

This is the complete list of members for jaligner.Alignment, including all inherited members.

|  |  |  |
| --- | --- | --- |
| add(Alignment a1, Alignment a2) | jaligner.Alignment | static |
| Alignment() | jaligner.Alignment |  |
| calculateScore() | jaligner.Alignment |  |
| checkScore() | jaligner.Alignment |  |
| copy(Alignment alignment) | jaligner.Alignment | static |
| GAP | jaligner.Alignment | static |
| getExtend() | jaligner.Alignment |  |
| getGaps() | jaligner.Alignment |  |
| getGaps1() | jaligner.Alignment |  |
| getGaps2() | jaligner.Alignment |  |
| getIdentity() | jaligner.Alignment |  |
| getLength() | jaligner.Alignment |  |
| getLongestPerfectMatch() | jaligner.Alignment |  |
| getLongestUngappedAlignment(int maxMismatches) | jaligner.Alignment |  |
| getMarkupLine() | jaligner.Alignment |  |
| getMatrix() | jaligner.Alignment |  |
| getName1() | jaligner.Alignment |  |
| getName2() | jaligner.Alignment |  |
| getNumberOfMatches() | jaligner.Alignment |  |
| getNumberOfMatches2() | jaligner.Alignment |  |
| getOpen() | jaligner.Alignment |  |
| getOriginalSequence1() | jaligner.Alignment |  |
| getOriginalSequence2() | jaligner.Alignment |  |
| getPercentIdentity() | jaligner.Alignment |  |
| getScore() | jaligner.Alignment |  |
| getScoreWithNoTerminalGaps() | jaligner.Alignment |  |
| getSequence1() | jaligner.Alignment |  |
| getSequence2() | jaligner.Alignment |  |
| getSimilarity() | jaligner.Alignment |  |
| getStart1() | jaligner.Alignment |  |
| getStart2() | jaligner.Alignment |  |
| getSummary() | jaligner.Alignment |  |
| setExtend(float extend) | jaligner.Alignment |  |
| setGaps(int gaps) | jaligner.Alignment |  |
| setIdentity(int identity) | jaligner.Alignment |  |
| setMarkupLine(char[] markupLine) | jaligner.Alignment |  |
| setMatrix(Matrix matrix) | jaligner.Alignment |  |
| setName1(String name1) | jaligner.Alignment |  |
| setName2(String name2) | jaligner.Alignment |  |
| setOpen(float open) | jaligner.Alignment |  |
| setOriginalSequence1(Sequence originalSequence1) | jaligner.Alignment |  |
| setOriginalSequence2(Sequence originalSequence2) | jaligner.Alignment |  |
| setScore(float score) | jaligner.Alignment |  |
| setSequence1(char[] sequence1) | jaligner.Alignment |  |
| setSequence2(char[] sequence2) | jaligner.Alignment |  |
| setSimilarity(int similarity) | jaligner.Alignment |  |
| setStart1(int start1) | jaligner.Alignment |  |
| setStart2(int start2) | jaligner.Alignment |  |
| toString() | jaligner.Alignment |  |


---

Generated on Fri Aug 15 2014 15:11:05 for ESAT by  

 1.8.7
